# Editorial Comment: Comparative Cost-effectiveness of Surgery, *Collagenase Clostridium Histolyticum*, and Penile Traction Therapy in Men with Peyronie's Disease in an Era of Effective Clinical Treatment

**DOI:** 10.1590/S1677-5538.IBJU.2020.01.08

**Published:** 2020-01-13

**Authors:** K Wymer, T Kohler, L Trost, Rodrigo R. Vieiralves

**Affiliations:** 1 Mayo Clinic Department of Urology Department of Urology, Mayo Clinic, Rochester, MN, USA; 2 Mayo Clinic Department of Urology Department of Urology, Mayo Clinic, Rochester, MN, USA; 1 Serviço de Urologia, Hospital Federal da Lagoa, Rio de Janeiro, RJ, Brasil

## COMMENTS

Peyronie's Disease has prevalence rates of 0.4-9%, with a higher prevalence in patients with ED and diabetes (1). We know that surgery has been considered the gold standard treatment of Peyronie's disease. In most countries we have found that conservative treatment has poor acceptability especially in patients with greater curvature (2–4). However, as we know, the cost of surgery is high, so that patients who do not have access or do not want this treatment have few or no alternatives. In this scenario, the comparison of cost effectiveness between conservative and surgical treatments gains importance and this is the subject of this interesting article.

In this paper, Dr Kevin Wymer and his colleagues from Mayo Clinic, compared the cost effectiveness of treatment with collagenase Clostridium Histolyticum (CCH), traction therapy (RXPTT- a novel penile traction therapy device) and surgery. To evaluate effectiveness, parameters such as an improvement of >20% in penile curvature, complications for each of the treatments and quality of life parameters were considered. After comparison and statistical analysis, he noted that because of the high cost of surgery, with higher complication rates such as erectile dysfunction as well as the high cost of CCH with some local complications (penile ecchymosis, penile hematoma), the total cost for providing the same increase in quality of life was smaller with the use the RXPTT, demonstrating that this non-surgical device appears to have place in the treatment of Peyronie's disease.

Some limitations were highlighted by the author. The success criteria were 20% curvature improvement without considering other aspects such as the final penile length. Another important point is the patient's expectation. If a patient's primary goal was to achieve a fully straight penis, surgery would be the most cost-effective option. In contrast, if length and preservation of erectile function were the primary objective, RXPTT would be preferred.

Some other aspects deserve to be mentioned. First, traction devices require treatment for a few hours during the day for a long period of time which decreases treatment adherence (5). Second, the choice of CCH as a comparison treatment: despite its proven effectiveness, we know it has a high cost (mean cost per patient = $ 33,628 at 10 years treatment) thus obviously disadvantaging in a cost effectiveness study. Also, other therapies such as injectable interferon, verapamil, oral pills and even vacuum devices should have been considered in the study. Finally, a point that needs to be commented even though noting that the study has been approved by the Mayo Clinic Conflict of Interest Board, is the fact that one of the authors of the paper is the developer of RXPTT, the device used in the study.

To conclude, we have here an unprecedented study on cost-effectiveness in Peyronie's disease, performed with methodological rigor, comparing three possible treatment modalities and which presents a new device for non-surgical treatment of this disease.
